# Smoking cessation after an acute coronary syndrome: immediate quitters are successful quitters

**DOI:** 10.1007/s12471-015-0755-9

**Published:** 2015-10-08

**Authors:** M. Snaterse, W.J.M. Scholte op Reimer, J. Dobber, M. Minneboo, G. ter Riet, H.T. Jorstad, S.M. Boekholdt, R.J.G. Peters

**Affiliations:** 1 University of Applied Sciences, Hogeschool van Amsterdam, Amsterdam School of Health Professions, Tafelbergweg 51, 1105 BD Amsterdam, The Netherlands; 2 Department of Cardiology, Academic Medical Center, Amsterdam, The Netherlands; 3 Department of General Practice, Academic Medical Center, University of Amsterdam, Amsterdam, The Netherlands

**Keywords:** Acute coronary syndrome, Smoking cessation, Secondary prevention

## Abstract

**Background:**

Cardiovascular disease (CVD) prevention guidelines stress the importance of smoking cessation and recommend intensive follow-up. To guide the development of such cessation support strategies, we analysed the characteristics that are associated with successful smoking cessation after an acute coronary syndrome (ACS).

**Methods:**

We used data from the Randomised Evaluation of Secondary Prevention for ACS patients coordinated by Outpatient Nurse SpEcialists (RESPONSE) trial (*n* = 754). This was designed to quantify the impact of a nurse-coordinated prevention program, focusing on healthy lifestyles, traditional CVD risk factors and medication adherence. For the current analysis we included all smokers (324/754, 43 %). Successful quitters were defined as those who reported abstinence at 1 year of follow-up.

**Results:**

The majority of successful quitters quit immediately after the ACS event and remained abstinent through 1 year of follow-up, without extra support (128/156, 82 %). Higher education level (33 vs. 15 %, *p* < 0.01), no history of CVD (87 vs. 74 %, *p* < 0.01) and being on target for LDL-cholesterol level at 1 year (78 vs. 63 %, *p* < 0.01) were associated with successful quitting.

**Conclusion:**

The majority of successful quitters at 1 year stopped immediately after their ACS. Patients in this group showed that it was within their own ability to quit, and they did not relapse through 1 year of follow-up. Our study indicates that in a large group of patients who quit immediately after a life-threatening event, no relapse prevention program is needed.

## Introduction

Coronary heart disease (CHD) patients are at high risk of recurrent coronary events and mortality. Risk reduction strategies are therefore offered to patients with established CHD or other atherosclerotic cardiovascular disease (CVD). Smoking is known to be a major health risk factor [1, 2]. Smoking cessation after CHD is diagnosed is potentially the most effective preventive measure. It is associated with a 33 %-50 % reduction in risk of recurrent myocardial infarctions or cardiovascular death [3–5] and a life expectancy gain of 3 years after coronary artery bypass surgery [6]. Nevertheless, although smoking cessation is potentially the most effective CVD prevention strategy, quitting smoking is difficult and secondary prevention is suboptimal. Studies from Europe and the USA have shown that half of the patients continue to smoke despite a life-threatening event [7, 8]. Although the majority received personal advice to stop or were offered counselling, many were not able to quit [7, 9]. In general, surveys revealed a disappointing situation with regard to secondary prevention actions. A substantial potential to reduce the risk of recurrent cardiovascular disease or death still remains.

Successful strategies for smoking cessation include pharmacological therapy (nicotine replacement therapy, bupropion and varenicline) and behavioural counselling for smokers willing to quit [10–12] Successful behavioural support in smoking cessation has been reported for group therapy, individual counselling [11] and telephone counselling [13] and to a lesser extent for individually tailored self-help materials [11]. In addition, guidelines on CVD prevention recommend frequent follow-up visits for all smokers who have quit to increase long-term success [14]. However, as shown in a recent review [15], the effectiveness of behavioural relapse prevention methods for any initially successful subgroup of former smokers has not been demonstrated.

Nurse-coordinated prevention programs also aim to increase the proportion of patients achieving CVD prevention targets, but these initiatives have not resulted in higher smoking cessation rates [16–19]. With the RESPONSE trial we evaluated whether a nurse-coordinated prevention program leads to better achievement of guideline-recommended CVD prevention targets [20]. We found this program improved blood pressure and lipid management, but did not have a significant impact on lifestyle factors, including smoking cessation [20].

It is currently unknown which patients benefit from intensive smoking cessation counselling after hospital admission for acute coronary syndrome (ACS). Better understanding of the characteristics of patients who are likely to quit successfully after ACS may provide useful information to guide development of more effective smoking cessation interventions. We therefore addressed the following research question: what are the characteristics of successful quitters after a recent ACS?

## Methods

### Design and study population

The RESPONSE trial (*n* = 754) was a multicentre, randomised controlled trial conducted in 11 centres in the Netherlands with 1 year of follow-up. Patients aged 18–80 years were eligible if they had been diagnosed and hospitalised with ACS within 8 weeks prior to enrolment in the trial. Patients were excluded if they (1) were unable to visit the nurse-coordinated prevention program, (2) were not available for follow-up, (3) had a limited life expectancy (≤ 2 years), and (4) were diagnosed with a New York Heart Association class III or class IV heart failure. Patients were randomised to either the nurse-coordinated prevention program or usual care alone. Detailed information about the study methods has been reported elsewhere [20, 21]. For the current analyses, we selected 324 patients who smoked before the index ACS event (43 %) and reported a smoking and quitting status at 1 year of follow-up.

We defined successful quitters as patients who reported abstinence accompanied by a quit date at 1 year of follow-up. We defined relapsers as those patients who had attempted to quit smoking but reported that they began smoking again within 1 year, and were therefore classified as ‘smoker’ in the main analysis at 1 year of follow-up. Patients who reported that they continued smoking in the year of follow-up were also classified as smokers.

### Data collection and follow-up

Baseline measurements were performed within 8 weeks after ACS. Patients were enrolled at an average of 4 weeks (SD 2.7). Patients in the intervention group visited the outpatient clinic four times during the first 6 months after inclusion, in addition to visits to their treating cardiologist (usual care). During each nurse visit cardiovascular risk factors were evaluated. Data on clinical and demographic characteristics, CHD risk factors and smoking quit dates were collected at baseline and follow-up. Smoking behaviour was measured by means of interview questions. Health-related quality of life was assessed with the MacNew questionnaire [22, 23]. Scores on each quality of life domain were calculated as the average of the responses in that domain. We used the Systematic Coronary Risk Evaluation (SCORE) as an integrated measure to estimate the overall impact of smoking cessation on cardiovascular risk.

### Statistical analysis

The results of our statistical analysis are presented as absolute numbers and percentages. Differences between successful quitters and smokers were analysed by using unpaired t-tests for continuous variables and chi-square statistics for categorical variables. We used SPSS (version 20.0) for all data analysis.

## Results

**Fig. 1:**
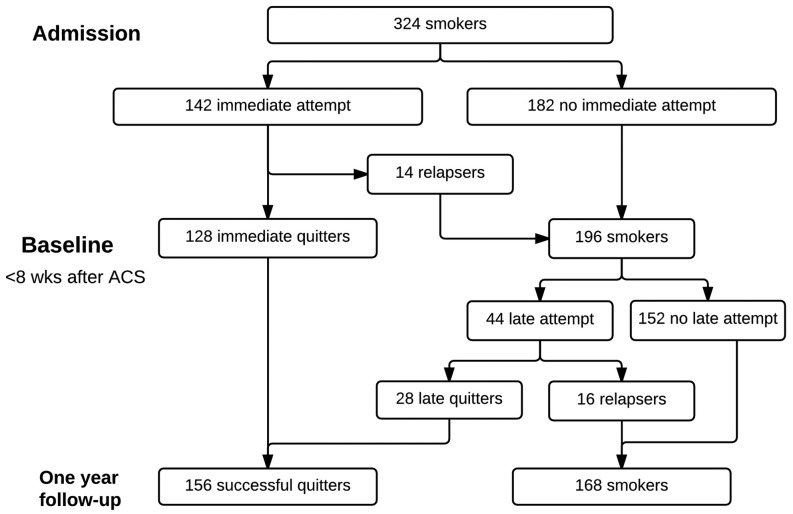
Flowchart of 324 smokers after an acute coronary syndrome (ACS) from hospital admission up to 1-year follow-up

Of 324 smokers admitted to hospital with ACS, 186 (57 %) reported a cessation attempt in the year after the event. Of those, 156 (86 %) were successful quitters in up to 1 year of follow-up. The majority of this group quit immediately after the event (128/156, 82 %; Fig. 1) and received no smoking cessation counselling after discharge. Patients making a later cessation attempt were less successful in quitting smoking (28/44, 64 %).

As shown in Table 1, successful quitting up to 1 year after ACS was associated with a higher education level (33 vs. 15 %, *p* < 0.01), no history of CVD (87 vs. 74 %, *p* < 0.01), being on target for LDL-cholesterol level at 1 year (78 vs. 63 %, *p* < 0.01) and adequate physical activity at 1 year (65 vs. 52 %, *p* = 0.01).

**Table 1:** Characteristics of successful quitters versus smokers in ACS patients (*n* = 324) Immediate: immediately after hospital discharge; late: during one year of follow-up.

	Successful quitters^a^ *n* = 156	Smokers *n* = 168	*P*-value^b^	Relapsers^c^ *n* = 30
Age				
< 50 years	53 (34 %)	61 (36 %)	0.89	14 (47 %)
50–59 years	67 (43 %)	71 (42 %)		13 (43 %)
≥ 60 years	36 (23 %)	36 (21 %)		3 (10 %)
Male, *n* (%)	127 (81 %)	125 (74 %)	0.13	20 (67 %)
Highest level of education, *n* (%)				
Fewer than 8 years	41 (28 %)	63 (38 %)	0.02	13 (43 %)
College or university	49 (33 %)	25 (15 %)	< 0.001	5 (17 %)
No history of CVD, *n* (%)	136 (87 %)	124 (74 %)	< 0.01	19 (63 %)
Index event, *n* (%)				
STEMI	89 (57 %)	89 (53 %)	0.89	18 (60 %)
NSTEMI	50 (32 %)	51 (30 %)		7 (23 %)
Unstable angina pectoris	17 (11 %)	26 (16 %)		4 (13 %)
Nurse Coordinated Prevention Programme	89 (57 %)	83 (49 %)	0.17	16 (53 %)
No. cigarettes/day				
≤ 10	62 (40 %)	59 (35 %)	0.36	10 (33 %)
> 10	93 (60 %)	109 (65 %)		20 (67 %)
Quality of life at baseline^d^ (mean, SD)	5.13 (1.06)	5.02 (1.14)	0.47	5.0 (0.9)
Quality of life at 1-year follow-up	5.66 (1.01)	5.46 (0.99)	0.66	5.6 (0.7)
**Risk factors at baseline**				
Systolic blood pressure > 140 mmHg	36 (24 %)	33 (20 %)	0.12	4 (13 %)
LDL-cholesterol > 2.5 mmol/L	46 (31 %)	66 (39 %)	0.15	13 (43 %)
Body mass index > 25 kg/m^2^	116 (74 %)	115 (68 %)	0.12	22 (73 %)
Inadequate physical activity^e^	89 (57 %)	98 (58 %)	0.81	16 (53 %)
**Risk factors at 1-year follow-up**				
Systolic blood pressure > 140 mmHg	41 (28 %)	43 (26 %)	0.79	9 (30 %)
LDL-cholesterol > 2.5 mmol/L	32 (22 %)	62 (37 %)	< 0.01	16 (57 %)
Body mass index > 25 kg/m^2^	127 (81 %)	112 (67 %)	< 0.01	23 (78 %)
Inadequate physical activity	54 (35 %)	81 (48 %)	0.01	15 (50 %)
Systematic Coronary Risk Evaluation (SCORE)	2.9 %	5.7 %	< 0.01	4.2 %

^a^Defined as non-smoking at outcome assessment date;

^b^Between successful quitters and smokers;

^c^note that these 30 relapsers are a subgroup of the 168 smokers;

^d^ *assessed with the MacNew questionnaire;*

^e^< *30 min/5 times a week.*

At 12 months, the estimated SCORE cardiovascular 10-year mortality risk was 2.9 % (SD 0.03) for successful quitters and 5.7 % (SD 0.07) for smokers (*p* < 0.01). Successful quitters and smokers were comparable in other lifestyle risk factors than smoking at baseline, while after 1 year successful quitters more frequently had a body mass index (BMI) > 25 kg/m^2^ compared with smokers (81 vs. 67 %, *p* < 0.01) Mean BMI at 1 year was 29.0 kg/m^2^ (SD 4.93) in successful quitters and 27.5 kg/m^2^ (SD 5.04) in smokers. Smoking cessation after ACS was associated with an average weight gain of 3.36 kg (SD 5.48) at 1 year. In our study we observed a maximal weight gain in successful quitters of 21 kg, whereas 9 % of successful quitters gained > 10 kg after smoking cessation.

Within the group of smokers, 63 % reduced smoking cigarettes at 1 year of follow-up compared with baseline level. These patients had a higher level of education and smoked a higher number of cigarettes per day compared with smokers not reducing cigarette smoking. We observed a median reduction of 5 (IQR 0–15) cigarettes for smokers at 1 year of follow-up. In smokers who reduced cigarette smoking, we found that after 1 year they smoked a median of 13 cigarettes (IQR 6–20) less than at baseline.

In total, 30 relapsers were presented (Table 1). The majority (90 %) of these were younger than 60 years of age, relatively more were female (33 %) than both successful quitters and smokers and diagnosed with ST-segment-elevation myocardial infarction (60 %). We found that a group of 14 relapsers before baseline measurements were predominantly male coronary artery bypass graft (CABG) patients (86 %).

Of 44 patients making later cessation attempts, 73 % were in the nurse-coordinated prevention program group. This group was encouraged to quit smoking and was given information about a healthy lifestyle. However, participation in the nurse-coordinated prevention program group did not significantly increase smoking cessation rates in patients making a late attempt (*p* = 0.8).

## Discussion

Our study demonstrates that immediate cessation after hospitalisation for ACS is the most important characteristic of successful quitters. A higher level of education, no history of CVD, LDL-cholesterol level on target and adequate physical activity at 1 year characterised successful quitters at 1 year after ACS as well. At 1 year, however, successful quitters more often had a BMI > 25 kg/m^2^ compared with smokers.

The REPONSE trial showed that a nurse-coordinated prevention program improved systolic blood pressure and blood levels of LDL-cholesterol. However, this program was less successful in achieving smoking cessation [20]. In the current paper we explored characteristics that may increase successful smoking cessation, for smoking is a major risk factor of mortality and recurrent events in CHD patients.

Our study confirms that quitting smoking is extremely difficult for many patients, even after being hospitalised for a life-threatening event, especially for those with a lower education level. Only half of the patients succeeded in quitting smoking after ACS, which is consistent with success rates in previous studies [7, 9].

On the positive side, however, our study also shows that almost half of all smokers succeeded in quitting up to 1 year after ACS. Moreover, of those who quit immediately after the acute event, the majority are successful through 1 year. Our study confirms earlier findings indicating that a clinical event acts as an important motivator and may induce behavioural change [24], particularly if this event is perceived as life-threatening as is the case with patients’ first ACS [9, 25]. In accordance with European Society of Cardiology (ESC) guidelines, clinicians may make greater use of this opportunity by addressing the issue before discharge [14]. These guidelines also recommend that support for cessation of smoking is initiated for all smokers during hospital admission and is continued for a prolonged period after discharge [10, 14]. Our study shows, however, that the majority of successful quitters stop immediately after discharge, triggered by the ACS event, and that it is within their own ability to quit and remain abstinent.

This continued change of behaviour may be explained by the theory of self-perception, which describes how people use their own behaviour to learn what they believe [24]. In our study, during admission almost half of the smokers showed that they were willing to change and felt able to change. The feeling of being able to change is strengthened when these patients indeed quit smoking after discharge. These patients soon perceive themselves as ‘successful quitters’ [24], which subsequently strengthens them in their resolve to remain abstinent. Moreover, for these patients- who are in a ‘ready for action’ stage, according to the stages of change theory of Prochaska and Diclemente [26] - counselling seems unnecessary and may be even counterproductive [24, 27]. The results of our study therefore suggest that the WHO smoking cessation algorithm, which is included in the ESC guideline and recommends intensive follow-up for all smokers, may not be appropriate for smokers who quit immediately after ACS. In the decision-making process about smoking cessation interventions, a distinction could be made between types of smokers, such as quitters triggered by an acute life-threatening event or other triggers and immediate or late quitters. In patients hospitalised for acute events who immediately quit after discharge, and do not relapse up to their first outpatient clinic visit, relapse prevention by counselling or pharmacological therapy may not be necessary. In our study, none of the immediate quitters who remained abstinent up to their first outpatient visit reported a relapse up to 1 year after ACS, and evidence for the effectiveness of relapse prevention for patients who immediately quit smoking after an acute hospitalisation is lacking [15].

Our results are, however, less clear about the effectiveness of smoking cessation interventions at hospital discharge, as we observed a number of relapsers between hospital discharge and the first visit to the outpatient clinic. This occurred particularly in CABG patients, who may feel the external pressure not to smoke, but may not be intrinsically motivated to quit or not feeling able to quit. Smoking reduction to support smoking cessation could be considered for smokers who are willing but unable to quit [28]. Reduced smoking can be advised until these patients are ready for a new attempt [28, 29]. More research is needed on characteristics of ACS patients who intend to quit smoking during hospitalisation, in order to focus on those who are willing to quit but are at risk for relapse after discharge. Our study confirms earlier research showing that successful quitting is strongly associated with a higher education level and no history of CVD [9, 25]. Deferral of smoking cessation interventions after hospital discharge should be considered in patients with these characteristics. In our study, pharmacological support was not part of the smoking cessation counselling, although guidelines recommend offering aids to assist cessation. Nicotine replacement therapy, bupropion and varenicline have been shown to improve the chances of successful quitting, although patients with a recent history of cardiac disease were excluded in these studies [12, 30] Therefore, the results of pharmacological studies may not be applied to immediate quitters after an acute event. Regarding relapse prevention, the only medical therapy for which there is compelling evidence is varenicline [15]. More research is needed on the effectiveness of pharmacological aids in immediate quitters after an acute life-threatening event.

Furthermore, since nurses obtained information on smoking quitting dates retrospectively, recall bias may play a role. Successful quitters may remember quitting dates better than smokers who attempted to quit but relapsed. In addition, we may have underestimated the problem of unsuccessful quit attempts, as self-report information is less reliable than measurements of nicotine concentration [31].

Lastly, we observed that successful quitters have an unfavourable risk factor profile after 1 year. Consistent with previous reports, quitters were more often overweight [32, 33]. We also observed that quitters did not improve on systolic blood pressure targets at 1 year of follow-up compared with their baseline values, despite the fact that they reported being physically more active. It is well known that quitting smoking decreases the metabolic rate, which results in a mean increase of 4–5 kg in body weight after 12 months [14, 32, 33]. Moreover, some patients exchange one addiction for another, and gain weight after quitting smoking [34]. In fact, the addiction may not be interrupted, but simply replaced by another [34]. Future research is needed to investigate the mechanisms involved and to improve weight loss interventions for this subgroup.

We conclude that the majority of successful quitters stop immediately after their ACS. Patients in this group showed that it was within their own ability to quit, and they did not relapse in 1 year of follow-up. We found no evidence to support the use of relapse prevention in ACS patients who stop smoking immediately after the event, and our study indicates that there is no need for this during follow-up visits in a large group of patients. The momentum for smoking cessation is particularly strong immediately after ACS and our study reinforces the importance of clinicians’ explicit advice to stop smoking during hospitalisation of ACS patients. New strategies are needed in patients with a late attempt. Smoking cessation strategies in secondary prevention could differentiate between acute and non-acute patients, since an acute event acts as an important motivator for behavioural change. Furthermore smoking cessation support should differentiate between immediate and late attempts, since relapse prevention seems unnecessary for immediate quitters. However, patients with a late attempt may benefit from more intensive therapy. Future research is needed to assess the cost-effectiveness of differentiating between acute or non-acute admissions and immediate or late quit attempts.

### Funding

The RESPONSE trial was sponsored by an unrestricted grant from AstraZeneca, the Netherlands. The sponsor had no role in the design, data collection, data analysis, data interpretation and writing of this report.

### Conflicts of interests

M. Snaterse, W.J.M. Scholte op Reimer, M. Minneboo, J. Dobber, G. ter Riet, H.T. Jorstad, S.M. Boekholdt and R.J.G. Peters declare no conflict of interest.
